# Tacrolimus-Associated Hyperpigmentation: A Case Report and Literature Review

**DOI:** 10.7759/cureus.92656

**Published:** 2025-09-18

**Authors:** Bryan Tassavor, Rishi Chopra

**Affiliations:** 1 Osteopathic Medicine, A.T. Still University School of Osteopathic Medicine, Mesa, USA; 2 Dermatology, UnionDerm Private Practice, New York, USA

**Keywords:** drug-induced hyperpigmentation, pediatric case report, pigmentary changes, topical tacrolimus, vitiligo

## Abstract

Topical tacrolimus is an immunomodulator commonly used in the management of vitiligo. We report a case of tacrolimus-associated hyperpigmentation in a nine-year-old girl with non-segmental vitiligo, who presented with depigmented patches on the eyelid and trunk. The depigmented eyelid patch improved with topical tacrolimus but developed localized, paradoxical hyperpigmentation at the treated site. She was managed with alternating topical corticosteroids and tacrolimus alongside photoprotection; after six weeks, the trunk demonstrated full repigmentation, but the eyelid lesion developed marked hyperpigmentation temporally associated with tacrolimus use. Other causes of pigmentation were excluded, and the discoloration resolved completely within six weeks of discontinuing treatment. To further examine this presentation, we reviewed the literature for reports of similar pigmentary changes associated with tacrolimus. This review encompassed both oral and topical use in patients with and without vitiligo.

## Introduction

Vitiligo is characterized by decreased or absent melanocytes in the epidermis, manifesting clinically as well-demarcated depigmented macules and patches. These macules and patches often appear on the hands, forearms, feet, and face and may appear in a segmental, localized, or generalized, non-segmental distribution [1]. Vitiligo develops through immune-mediated destruction of melanocytes, but the underlying mechanisms differ between its subtypes. In non-segmental vitiligo, oxidative stress within melanocytes leads to the release of danger signals and activation of stress pathways that recruit CD8+ T cells, resulting in immune-driven melanocyte loss across widespread areas of skin [2-5]. In segmental vitiligo, by contrast, a localized immune attack against genetically mosaic melanocytes produces unilateral, sharply demarcated patches that typically appear rapidly and then stabilize [6-8]. Vitiligo has an unpredictable disease course with roughly half of all cases manifesting in childhood and adolescence [9]. Management of the condition primarily consists of topical corticosteroids, nonsteroidals such as tacrolimus, and energy-based devices such as lasers and phototherapy. Treatments often need to be continued indefinitely, as resident memory CD8+ T cells have been shown to develop and maintain melanocyte attack long-term in some patients [10].

Tacrolimus is a nonsteroidal immunomodulatory agent known for its efficacy in treating inflammatory and autoimmune conditions as well as serving as a mainstay in organ transplant rejection prevention. Tacrolimus binds to cytosolic FK506-binding proteins (FKBPs), inhibiting calcineurin activation of nuclear factor of activated T cells (NFAT) and inhibiting T-cell proliferation, a key factor in many inflammatory and autoimmune disorders [11]. Despite not being FDA-approved for vitiligo, topical tacrolimus is believed to be efficacious in inducing repigmentation in pediatric and adult patients. Randomized trials and meta-analyses show that topical tacrolimus can achieve ≥75% repigmentation in up to 65% of adults with facial vitiligo, with systematic reviews reporting at least mild repigmentation in over half of patients with the best responses on the face and neck [12,13]. Beyond inducing repigmentation, tacrolimus also reduces relapse risk when used as maintenance therapy and has demonstrated benefit in acral disease by limiting new lesions and enhancing repigmentation [14,15]. There is also specific data to support its use in pediatric populations with facial lesions [16]. The topical formulation of tacrolimus in the treatment of vitiligo is generally well tolerated with the most common adverse effects associated with its application being burning, pruritus, and erythema. A lesser-known complication of tacrolimus treatment for vitiligo, however, is hyperpigmentation. We present one such case and a subsequent review of the literature.

## Case presentation

**Figure 1:**
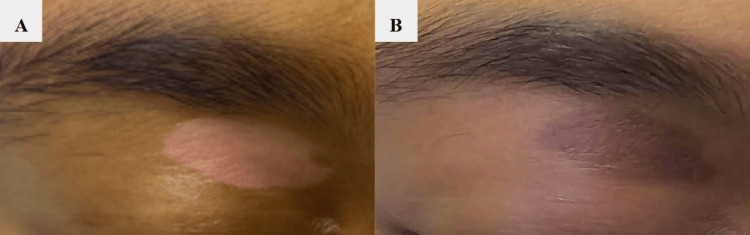
Clinical photos of topical tacrolimus hyperpigmentation reaction. A: Supraorbital hypopigmented vitiligo lesion prior to tacrolimus treatment. B: New onset hyperpigmented lesion after treatment

A nine-year-old female patient of South Asian descent presented in August 2024 with a several-month history of well-circumscribed depigmented patches with sharply demarcated borders on the right upper eyelid and posterior trunk (Figure 1A). There was no reported family history of vitiligo, autoimmune disorders, or atopy. The patient was diagnosed with non-segmental vitiligo and treated with an alternating regimen of two weeks of twice-daily hydrocortisone 2.5% cream and two weeks of twice-daily topical tacrolimus 0.03% ointment for the eyelid. For the trunk, a nearly identical regimen was used with mometasone 0.1% cream instead of hydrocortisone. The patient was counseled on photoprotection throughout the regimen.

After six weeks of treatment, the patient returned with full repigmentation of the trunk but marked hyperpigmentation confined to the right upper eyelid lesion (Figure 1B). The lesion appeared as a hyperpigmented patch, similar in size and shape to the initial vitiligo lesion. The patient’s parent reported that the discoloration developed gradually and progressed throughout the follow-up period. Other potential causes of hyperpigmentation were considered and excluded. The patient had no prior history of acne or trauma in the affected area, such as insect bites, and was not taking medications commonly associated with drug-induced pigmentation, including minocycline, hydroxychloroquine, chloroquine, NSAIDs, or cytotoxic agents. Her parents denied use of additional topical treatments and confirmed consistent adherence to photoprotection. Blood work was deferred given the clear temporal association with tacrolimus. All treatment was discontinued, and after six weeks off tacrolimus, the hyperpigmentation had completely resolved without residual adverse effects. By November 2024, the patient demonstrated full resolution without recurrence.

## Discussion

There were 15 studies included in the review, comprising 49 total cases of tacrolimus-induced hyperpigmentation. Of these cases, 46 patients had been using topical tacrolimus, while three patients had been using oral tacrolimus. The findings of the review are documented in Table 1.

**Table 1:** A chronological summary of the findings in the relevant literature describing tacrolimus-induced hyperpigmented lesions. Study findings were organized by patient demographics, indications for treatment, treatment regimen and details regarding the onset and evolution of the observed lesions a NR: not reported; OLP: erosive oral lichen planus; AD: atopic dermatitis

Study Authors (Publication Year)	Age (yrs), Sex, Race of patient(s)	Indication for Tacrolimus	Tacrolimus ROA, Dose, Regimen	Length of Tacrolimus use	Location and Description of Lesion	Final Outcome
Uemoto et al. (1994) [17]	Two pediatric patients of unknown demographics	Liver transplant prophylaxis	Oral, 0.15 mg/kg twice daily	30 months	Pigmentation of the oral mucosa	NR^a^
Bunetel et al. (2003) [18]	14 months, female, African-American	Liver transplant prophylaxis	Oral, 1 mg twice daily	40 months (lesions appeared at 22 months)	Pigmented lesions on the buccal/ labial mucosa & the gingiva	Tacrolimus was replaced with cyclosporin at 40 months. Symptoms fully resolved 18 months later
Shen et al. (2004) [19]	45 years, female, NR	OLP^a^	Topical, 0.1%, twice daily	9 months	Brown reticulated patch on lower gingiva/labial mucosa/oral vestibule	Tacrolimus discontinued. Pigmented patch resolved two months later
Fricain et al. (2005) [20]	72 years, female, NR	OLP	Topical, 0.1%, twice daily	2 months (lesion appeared at 1 month)	Brown oral mucosal staining	Tacrolimus discontinued at two months after treating OLP with resolution of staining one month later. OLP symptoms resumed after discontinuing and tacrolimus was continued again leading to the reappearance of the staining
Hickey et al. (2005) [21]	4 years, male, Afro-Caribbean	AD^a^	Topical, 0.1%, twice daily	15 months (lesions appeared at 9 months)	Multiple lentigines around knees and ankles; primarily at locations of application	Tacrolimus discontinued with lentigines persisting as of 18 months
Hickey et al. (2005) [21]	7 years, female, NR	AD	Topical, 0.1%, twice daily	17 months (lesions appeared at 5 months)	Over 100 brown macules (biopsied and confirmed to be lentigines) at neckline, wrists, ankles, upper thighs and lower back; primarily at locations of application	Tacrolimus was discontinued with lentigines persisting unchanged at 12 months post discontinuation
Hickey et al. (2005) [21]	11 years, female, NR	AD	Topical, 0.1%, twice daily	48 months (lesions appeared at 42 months)	Multiple brown macules (biopsied and confirmed to be lentigines) over wrists, hands, upper thighs, knees and ankles; primarily at locations of application	Tacrolimus was discontinued with lentigines persisting at six months post discontinuation
Kim et al. (2007) [22]	45 years, female, Korean	Lichen sclerosus	Topical, 0.1%, twice daily	3 months	Brown pigmentation on right buccal cheek at site of application	NR
De et al. (2008) [23]	10 years, male, NR	Non-segmental vitiligo	Topical, 0.03%, once daily	4 months	Brown pigmentation of unilateral infraorbital region at application site	Pigmentation subsided one month following discontinuation of tacrolimus
Zattra et al. (2010) [24]	16 years, male, Caucasian	AD	Topical, 0.1%, twice daily (3 weeks), once daily (6 weeks)	12 months (lesions appeared at 9 months)	Labial melanotic macule on lower lip	Tacrolimus continued following initial discovery of macules with lesions persisting at 12 months
Zattra et al. (2010) [24]	25 years, female, Caucasian	AD	Topical, 0.1%, twice daily	3 months	Two labial melanotic macules on lower lip	Tacrolimus was discontinued and lesions remained unchanged at 6-month follow up
Castelo-Soccio et al. (2012) [25]	11 pediatric patients and 1 adult patient	AD, psoriasis, perioral dermatitis, inverse psoriasis and Netherton syndrome	NR, Use of tacrolimus OR pimecrolimus included in study but not specified	1 month - 7 years	Lentigines at various locations including sun-protected areas	Tacrolimus or pimecrolimus was discontinued in all patients with the lentigines partially regressing in 4/12 patients.
Sahni et al. (2014) [26]	10 years, male, NR	Non-segmental vitiligo	Topical, 0.03%, once daily	2 months	Hyperpigmented macules over bilateral periorbital vitiligo lesions	Tacrolimus was discontinued and pigmented patches were partially resolved on one-month follow up
Shi et al. (2014) [27]	40 years, female, Asian	Lip dermatitis	Topical, 0.1%, twice daily	2 weeks of tacrolimus followed by six months of pimecrolimus (topical 1%) (lesions appeared 2 months into treatment)	Greater than 10 melanotic macules on upper and lower lips	Macules continued to darken and increase in number for several months following discontinuation of treatment with calcineurin inhibitors. Patient lost to follow up
Gan et al. (2017) [28]	9 years, female, Caucasian	Non-segmental vitiligo	Topical, 0.1%, twice daily	82 months (lesions appeared at 72 months)	Lentigines in perioral area at application site	The patient was transitioned to tacrolimus use exclusively in the spring and summer months at 72 months. Despite continued improvement of the vitiligo lesions however, the lentigines remained. At 82 months, the patient was taken off of the tacrolimus for the following three months which resulted in resolution of the lentigines
León-Muiños et al. (2018) [29]	11 years, male, Caucasian	AD	Topical, 0.03%, twice daily	8 months	Lentigines on antecubital areas at application sites	Tacrolimus discontinued however lesions persisted with no improvement a year later
Deilhes et al. (2020) [30]	60 years, male, NR	Unspecified vitiligo	Topical, 0.1%, twice daily	3 months	Hyperpigmented patch on penis at area of application	Tacrolimus discontinued and the authors were able to successfully treat the hyperpigmentation with a 532 nm Q-switched laser after it failed to resolve upon tacrolimus discontinuation.
Seneschal et al. (2021) [12]	9 male patients and 11 female patients of a mean age of 47 years, ethnicity NR	Unspecified vitiligo	Topical, 0.1%, twice daily	24 weeks	Hyperpigmentation, unspecified	One patient developed hyperpigmentation by the end of the study.
Tassavor et al. (2024)	9 years, female, Indian	Non-segmental vitiligo	Topical tacrolimus, 0.03%, twice daily	6 weeks	Hyperpigmentation of right eyelid vitiligo lesion	After six weeks post tacrolimus discontinuation, the hyperpigmentation had fully resolved

Tacrolimus-induced hyperpigmentation is a relatively rare occurrence in the literature, relevant to both vitiligo patients on topical therapy and organ transplant patients taking it orally.

It is unclear whether the cases featuring hyperpigmentation from systemic tacrolimus treatment are due to the same etiology as those from topical treatment. Nor is it clear that hyperpigmentation from topical treatment of vitiligo occurs for the same reasons as in atopic dermatitis or lichen planus.

The liver transplant cases describe how long-term use of the medication can lead to oral mucosal hyperpigmentation. This is similar to the oral mucosal hyperpigmentation seen in the treatment of oral lichen planus. This contrasts somewhat with the lesional hyperpigmentation observed in vitiligo treatment and the appearance of lentigines in atopic dermatitis treatment.

In the vitiligo studies, two of the three reported cases of hyperpigmentation following tacrolimus treatment for hypopigmented vitiligo lesions involved the periorbital area. The prevalence of this occurrence in the region may be tied to a hypothesized increased efficacy of the medication in the area [13]. Additionally, unlike in our case, the patients in these studies had not been counseled on UV radiation exposure during tacrolimus treatment, raising the question of whether UV radiation could be a causative factor. These lesions appeared to resolve within a couple of months off treatment, much like in our case. Deilhes et al., however, successfully treated one apparently refractory case with the use of a 532 nm Q-switched laser [30].

In the lentigines studies, in contrast to the vitiligo studies, multiple small macules tended to appear at the sites of application after a prolonged period of use and did not respond to discontinuation of the treatment. The labial melanotic macule studies similarly depicted patients who presented with macules of varying sizes and patterns that failed to respond to discontinuation of the medication, possibly suggesting some difference in etiology between these presentations and the vitiligo ones.

Given these variable presentations, careful history-taking and exclusion of alternative causes of hyperpigmentation are essential before attributing new pigmentation to tacrolimus. Clinicians should specifically consider factors such as recent UV exposure, post-inflammatory changes, concurrent therapies, and underlying systemic disease. This is particularly important in vitiligo, where adjunctive phototherapy and background treatments are common, and where failure to account for these confounders could lead to misattribution.

The general mechanism behind how tacrolimus causes hyperpigmentation of any kind is still largely unknown. Earlier studies implicated keratinocyte modulation as well as tyrosinase activity as two key factors behind its ability to induce pigmentation [31]. More recently, the study by Jung et al. expands on this finding [32]. The study suggests that activated FK506 binding proteins are thought to indirectly promote the maturation and transfer of melanosomes. An interesting detail in the study by Jung et al. was that UVB-mediated melanosome secretion was increased by FK506 binding protein activation. This suggests sun exposure may contribute to tacrolimus-induced hyperpigmentation, though it likely did not play a role in the penile and labial hyperpigmentation cases noted earlier. The lichen sclerosus study by Kim et al. examined stem cell factor, a cytokine thought to be associated with melanocyte migration [22,31]. The authors hypothesized that topical tacrolimus was responsible for the increase in stem cell factor in dermal keratinocytes and fibroblasts in the biopsy findings of the patient in the study. This finding suggests that there may be a possible pathway to melanocyte activation through stem cell factor via topical tacrolimus. Bunetel et al. hypothesized in their organ transplant hyperpigmentation studies that the medication may have induced some sort of graft-versus-host disease-like reaction given the presentation, though no direct mechanism was hypothesized [18].

## Conclusions

It is important to recognize hyperpigmentation as a rare adverse effect of tacrolimus, particularly in vitiligo treatment. In cases of non-specific hyperpigmented lesions, such as in our case and in the majority of the reported vitiligo cases, our review shows that there is a reassuring trend of remission upon cessation of treatment. Separately, in many reported atopic dermatitis cases and in other cases, there are instances of the development of lentigines and labial macules that are more persistent. There is no clearly established mechanism in the literature describing the development of hyperpigmentation with tacrolimus use.
